# Description of *Telamoptilia grewiae* sp. n. and the consequences for the definition of the genera *Telamoptilia* and *Spulerina* (Lepidoptera, Gracillariidae, Gracillariinae)

**DOI:** 10.3897/zookeys.479.8899

**Published:** 2015-01-29

**Authors:** Tengteng Liu, Shuxia Wang, Houhun Li

**Affiliations:** 1 College of Life Sciences, Nankai University, Tianjin 300071, China

**Keywords:** Lepidoptera, *Telamoptilia*, new species, immature stage, leaf miner, China

## Abstract

The new species *Telamoptilia grewiae*, reared from leafmines on *Grewia biloba* (Malvaceae) is described with details on adult and immature stages. The larval head and the pupa are described for the first time in *Telamoptilia* Kumata & Kuroko, 1988, and are illustrated with scanning electron micrographs and line drawings. Photographs of adult habitus, wing venation, male and female genitalia, as well as host plant and mines are provided. The apomorphic adult and larval characters of the new species in *Telamoptilia* are discussed in relation to the recognition of the genera *Telamoptilia* and *Spulerina* Vári, 1961.

## Introduction

The genus *Telamoptilia* Kumata & Kuroko, 1988 is globally represented by five species that may be found in the Oriental and African regions. The type species *Telamoptilia cathedraea* (Meyrick, 1908) is geographically shared by the Oriental Region and Madagascar (De Prins and De Prins 2014). Three species are currently known from China, including *Telamoptilia cathedraea*, *Telamoptilia hemistacta* (Meyrick, 1924), and *Telamoptilia prosacta* (Meyrick, 1918).

The larvae of *Telamoptilia* species are leaf miners. Three plant families are known as hosts for *Telamoptilia*: Malvaceae, Amaranthaceae and Convolvulaceae (De Prins and De Prins 2014). Vári (1961) briefly described the biology of *Telamoptilia geyeri* (Vári, 1961). Kumata et al. (1988) described the biology and the larval body chaetotaxy of three species: *Telamoptilia cathedraea*, *Telamoptilia prosacta* and *Telamoptilia tiliae* (Kumata & Ermolaev, 1988). However, no larval head chaetotaxy and pupal features of *Telamoptilia* have been described so far.

*Telamoptilia grewiae* sp. n. is associated with Malvaceae and is described in the present paper from adult external characters, male and female genitalia, wing venation and immature stages. The larval head and pupal features are described for the first time in *Telamoptilia*.

## Methods

Field investigations were carried out in Mt. Baxian National Nature Reserves (40°11'N, 117°32'E), 300−600 m, Tianjin, China, from May to September in 2013 and June 2014. Leaves containing mines with larvae were placed in sealed plastic bags, or rearing containers with moist cotton. Larvae removed from mines were immersed in nearly boiling water for 30 seconds, and then were kept in 75% ethanol for morphological examination. Last instar larval skins, pupae, and exuviae were kept in 75% ethanol. Pupae in rearing containers were placed outdoors to overwinter, and were transferred into the laboratory at 20 °C on February 6, 2014. Emergence successively occurred from March 9 to early-April 2014. Adults were collected chiefly by rearing from immature stages, and occasionally by light trap.

Adult photographs were taken with a Leica M250A stereo microscope. Genitalia and wings were dissected and mounted according to the methods introduced by Li (2002), but stained with Eosin Y and/or Chlorazol Black, and the illustrations were prepared by using a Leica DM750 microscope, and refined in Photoshop® CS4 software. For scanning electron microscopy, larvae and pupae were dehydrated in gradient ethanol, dried in vacuum and coated with gold in a SCD 005 Sputter Coater (BAL-TEC), then operated with a voltage of 15 kV using Quanta 200 environmental scanning electron microscope (SEM) (FEI, Oregon). Line drawings were outlined from the photos taken by the Leica M250A stereo microscope, using path tool in Adobe Photoshop® CS4 software. Photographs of host plant, mines and a live adult were taken in the field using Canon PowerShot G10 digital camera.

Terminology of immature stages follows Davis and De Prins (2011) and De Prins et al. (2013), and that of adults follows Kumata et al. (1988). Thoracic segments I−III and abdominal segments 1−10 are abbreviated as TI−TIII and A1−A10, respectively.

All the specimens studied, including the types of the new species and the vouchered larvae and pupae, are deposited in the Insect Collection, Nankai University, Tianjin, China.

## Taxonomy

### Telamoptilia grewiae sp. n.

Taxon classificationAnimaliaLepidopteraGracillariidae

http://zoobank.org/584C70BC-8783-4E0E-A680-185E5EF7E1C7

Figures 1–6 , 7−9 , 10−13 , 14−19 , 20−23 , 24–32

#### Description.

**Figures 1–6.**
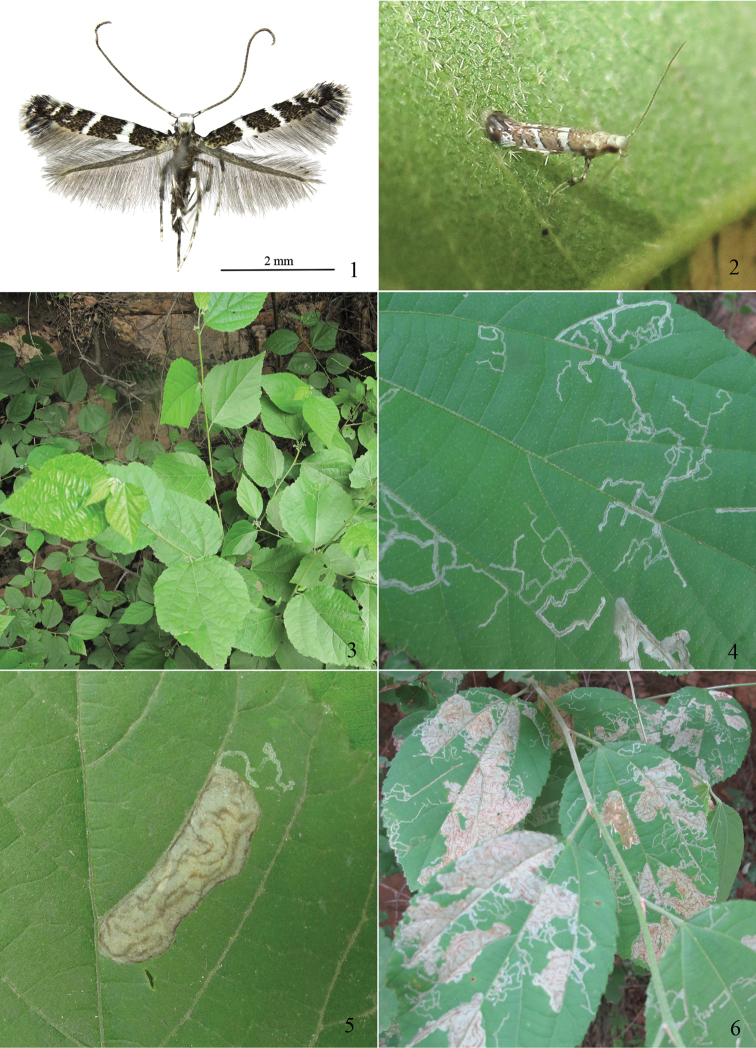
Adult, host plant and mines of *Telamoptilia grewiae* sp. n. **1** Adult in habitus, paratype **2** Live adult **3** Host plant **4** Linear mines by early instar larvae **5** Blotch mine by later instar larva **6** Seriously damaged leaves found in September.

Adults (Figs 1–2) with wing span 6.0−8.0 mm. Head silvery white, tinged with gray on face. Labial palpus grayish white, colored blackish gray on outer surface of distal half of second segment and before apex of third segment. Maxillary palpus white, with middle or distal half blackish fuscous. Antenna with scape white on posterior half, blackish gray on anterior half and distal portion, flap blackish gray tinged with white, as wide as scape in frontal view; flagellum silvery grayish fuscous, with each unit blackish distally. Thorax and tegula blackish gray mixed with white. Legs mostly white; foreleg with coxa blackish fuscous basally and distally, femur and tibia blackish fuscous, tarsus blackish gray distally on each except last segment; midleg with coxa blackish fuscous distally, femur blackish fuscous, except white medially and distally on dorsal surface, with ventral scale expansion blackish fuscous, tibia blackish fuscous basally and distally, white medially, tarsus white, each except last segment dotted blackish fuscous distally; hindleg with coxa blackish fuscous distally, femur blackish fuscous distally on outer surface, tibia blackish fuscous basally and distally, tarsus with basal three segments blackish fuscous distally, fourth segment dotted blackish fuscous dorso-distally. Forewing grayish fuscous to blackish fuscous; costal margin with a white spot basally at about 1/10 and one before apex, the former sometimes touching fold posteriorly, with white stria at distal 3/10 and 1/6 obliquely outward, reaching middle of wing and near termen respectively; transverse white fascia from costal 1/3 and 1/2 obliquely outward, reaching dorsal 1/2 and before end of fold respectively, edged with blackish fuscous to black scales, inner fascia wider than outer one, widened on posterior half; small white dot on distal end of M_3_, two or three small white dots along termen; apex blackish fuscous; cilia mostly blackish fuscous basally, gray distally, white adjacent to white markings, white on basal 1/4, black on median part, gray distally at apex, gray along dorsal margin. Hindwing and cilia uniformly gray.

**Variations.** The white costal spot at about basal 1/10 is sometimes reduced to a small dot near the costa, the white costal stria at distal 3/10 is occasionally extended to unite the dot on the distal end of M_3_, the costal stria at distal 1/6 is sometimes wedge-shaped or absent, the white dot at the distal end of M_3_ sometimes moves to near distal end of cell, and the small white dots along termen sometimes are absent.

**Figures 7–9.**
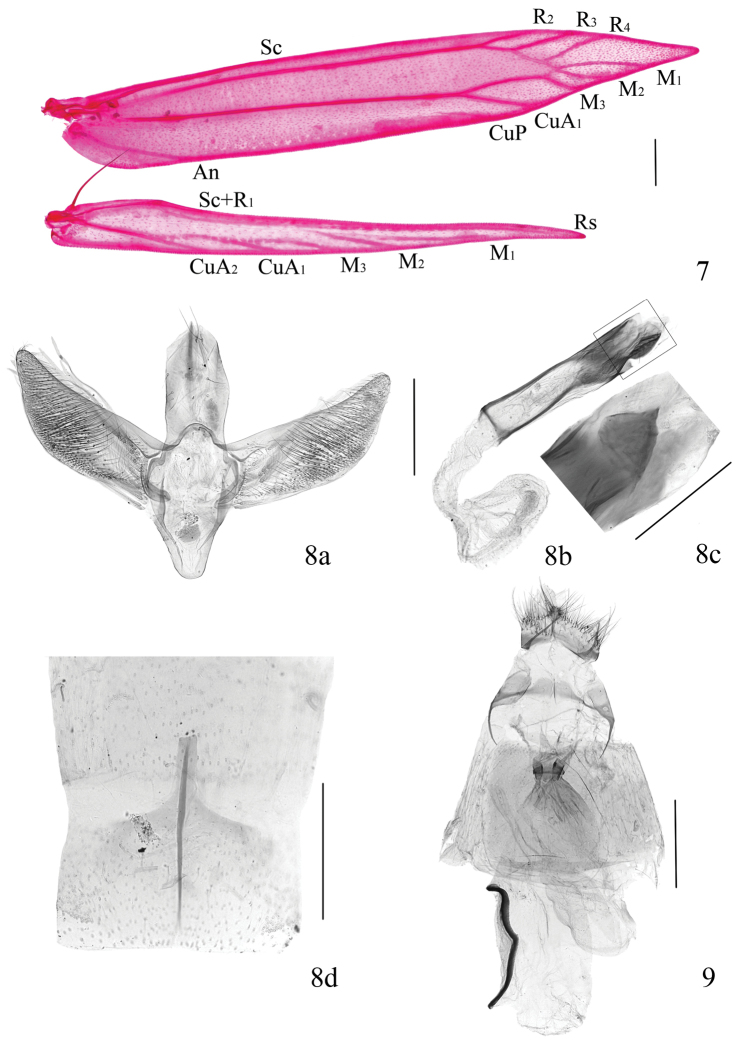
Wing venation and genitalia of *Telamoptilia grewiae* sp. n. **7** Wing venation, paratype, LTT12561W **8** Male genitalia, 8a, male genitalia with aedeagus detached, LTT12561 **8b** aedeagus, lateral view, same slide as **8a**, **8c** close-up of the distal portion of aedeagus, indicating the triangular process, dorsal view, LTT12257, 8d, eighth abdominal segment, paratype, same slide as 8a, **9** Female genitalia, paratype, LTT12555 (Scales = 0.2 mm except **8c** = 0.1 mm).

**Venation** (Fig. 7). Forewing with R_5_ totally untraceable, thus R_4_ not stalked, otherwise matching the generic characters (Kumata et al. 1988), with most notably the absence of R_1_.

**Male genitalia** (Figs 8a−d). Tegumen with basal half same width, median portion slightly widened, distal half almost triangular, rounded apically; three or four long setae along lateral side. Valva 1.7 times as long as tegumen, with basal 3/4 same width, distal 1/4 apparently narrowed, bluntly pointed apically. Saccus subtriangular, rounded apically. Aedeagus straight, almost as long as valva; distal half heavily sclerotized, with a triangular distal process about 1/8 length of aedeagus, pointed apically (Fig. 8c); vesica with dense spines becoming stronger towards distal (Fig. 8b). Eighth tergite with apodeme reaching posterior 1/3 of seventh segment, nearly parallel-sided (Fig. 8d).

**Female genitalia** (Fig. 9). Antrum a ring, disconnected ventrally, embed with a heavily sclerotized belt medially. Ductus bursae membranous, extremely short, not reaching anterior margin of seventh segment, wrinkled basally, without spines. Corpus bursae oval, membranous, without spines; signum slender and long, curved by 150° medially, posterior half slightly S-shaped, anterior half curved at anterior 2/5, sometimes slightly C-shaped.

**Last instar larva** (Figs 10–23). Length 4.0 mm, pale green to yellowish green. Three stemmata present (Fig. 11). Spiracles on TI and A8 larger; prolegs on A3−5 each with 2−4 crochets (Fig. 18), those on A10 without crochets. (Five larvae and two last instar larval skins examined)

**Figures 10–13.**
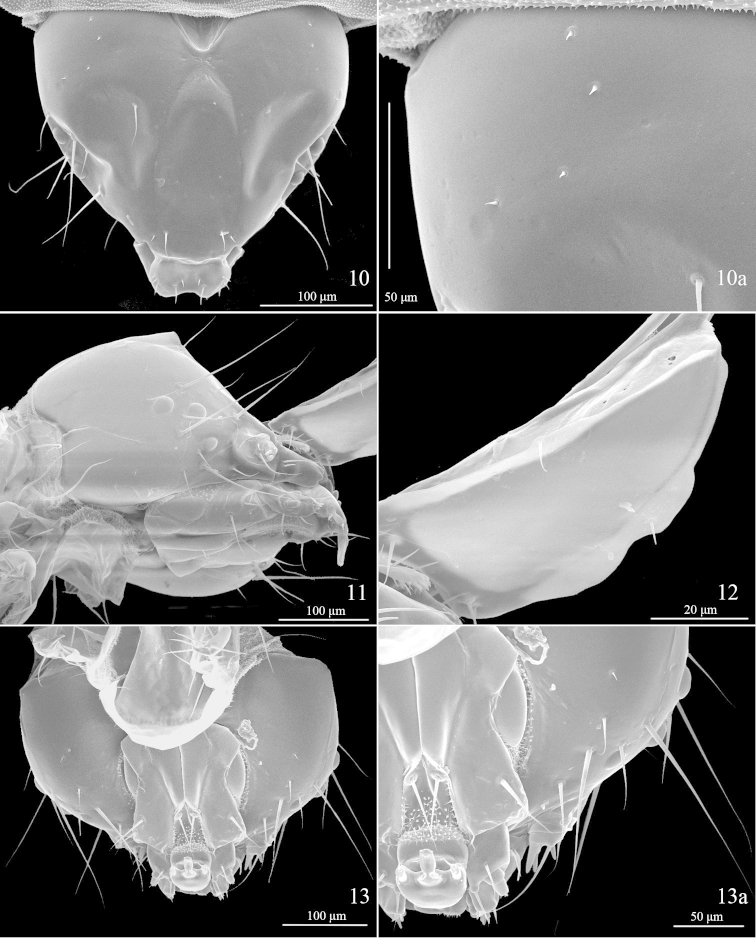
Last instar larva head chaetotaxy of *Telamoptilia grewiae* sp. n. **10** Dorsal view **10a** close-up of MD setae **11** Ventral-lateral view, scanned from last instar larval skin **12** Frons, frontal-lateral view, same individual as **11**, **13** Dorsal view **13a** close-up of SO and G setae.

**Figures 14–19.**
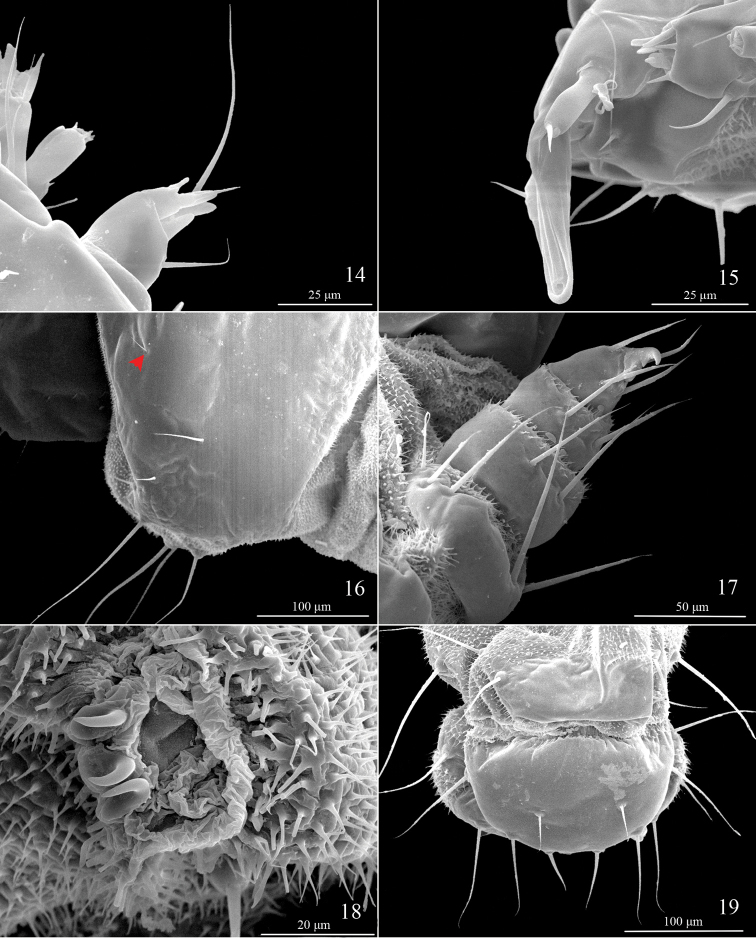
Last instar larval characters of *Telamoptilia grewiae* sp. n. **14** Antenna **15** Mouthpart **16** Setae on prothorax shield, arrow indicating the positon of XD1 **17** Thoracic leg **18** Proleg **19** A9−10, dorsal view.

**Figures 20–23.**
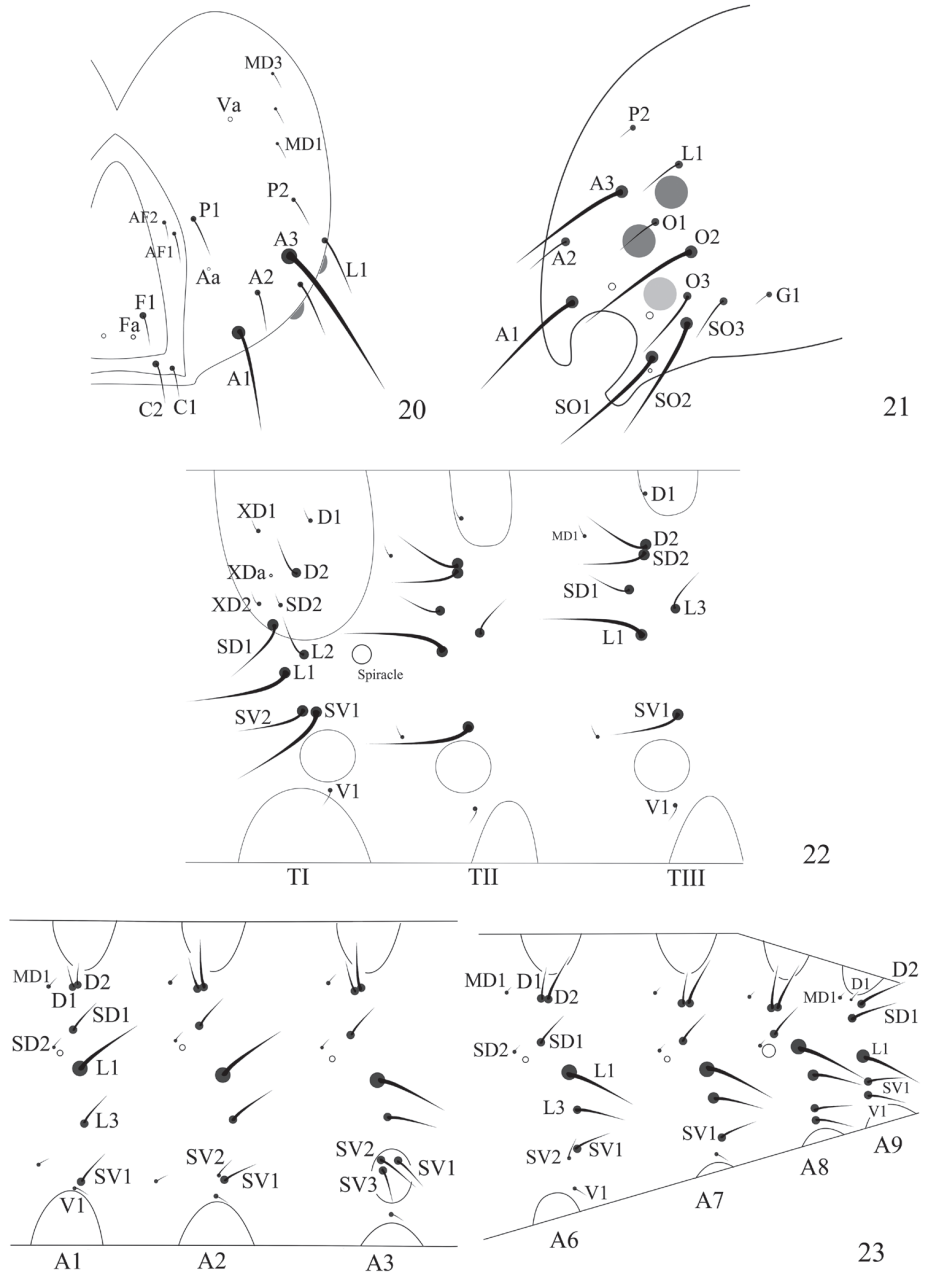
Line drawings of the larval chaetotaxy of *Telamoptilia grewiae* sp. n. **20** Head, frontal view **21** Head, lateral view **22** Thoracic segments, lateral view **23** Abdominal segments, lateral view.

*Head*. Adfrontal area slightly convex above middle, AF setae placed on convexity (Fig. 12); A3 longest, followed by A1, Aa internal to half way of A2 and A3, near margin of adfrontal area; P1 lateral to AF2, near margin of adfrontal area, P2 dorsal to A3, slightly shorter than P1; three MD setae placed posterior to P2, arranged in line, Pa internal and slightly anterior to MD2; O2 longest, followed by O3; SO1 as long as SO2, SO3 shorter, SOa near SO1; G1 posterior to SO3.

*Thorax*. TI with XD, D, and SD setae placed on prothoracic shield, XD1 near anterior margin of prothoracic shield (Fig. 16), XDa anterior to D2, SD2 near ventral margin of prothoracic shield; L-group bisetose, L2 dorsal and posterior to L1; SV-group bisetose, sharing pinaculum. TII with D1 near anterior dorsal margin, MD1 near posterior margin of prothoracic shield, anterior to D2; SD2 close to D2, SD1 anterior and ventral to SD2; L-group bisetose, L2 absent, L3 posterior and dorsal to L1; SV-group unisetose, with a micro seta anterior to SV1. TIII similar to TII.

*Abdomen*. A1−8 with D1 and D2 closely approximated to each other, MD1 seta anterior and slightly dorsal to D1, SD1 posterior and dorsal to spiracle, SD2 shorter than SD1, anterior and dorsal to spiracle, closer to spiracle than SD1, L-group bisetose, L2 seta absent; A1 and A7−9 with SV-group unisetose, A2 and A6 bisetose, A3−5 trisetose; A9 with D1 short, MD1 seta anterior to D1, SD1 anterior and ventral to D2, as long as D2, SD1, L1, SV1 and V1 almost forming a line; A10 as shown in Fig. 19.

**Figures 24–32.**
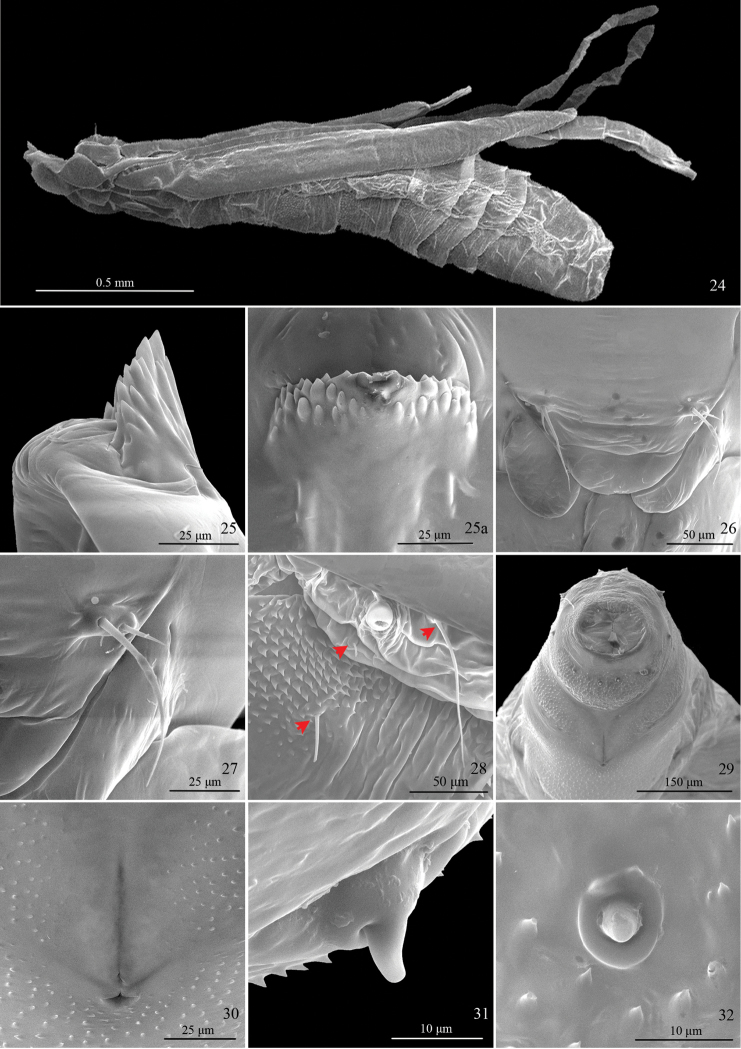
Pupal characters of *Telamoptilia grewiae* sp. n. **24** Pupa, lateral view **25** Frontal process (cocoon cutter), lateral view **25a** Frontal process (cocoon cutter), ventral view **26** Two pairs of sensillae on head **27** Close-up of the paired sensillae on head **28** Setae on A2, dorsal-lateral view, arrows indicating the positions of setae **29** Segments A9−10, ventral view **30** Genital orifice **31** Lateral cremaster, lateral view **32** Ventral cremaster, ventral view.

**Pupa** (Figs 24–32). Vertex with frontal process (cocoon cutter) triangular, densely covered with denticles dorsally, with 8−10 longitudinal grooves ventrally (Figs 25–25a); a pair of sensilla near base of each labial palpus, with inner sensillum three times longer than outer one (Figs 26–27); labial palpus 3.4 times longer than maxillary palpus. TII and TIII each bearing a pair of seta dorsally. A1−9 each carrying a pair of seta dorsally; A2−6 each bearing a pair of seta laterally, with one postero-ventral to spiracle, one internal to spiracle, shorter (Fig. 28); A2−10 densely covered with minute spines dorsally, which become stronger towards anterior margin on A2−7; cremaster with 9−11 denticles (Figs 29, 31–32). (Two preparations of exuviae and two pupae examined).

#### Diagnosis.

The new species mostly resembles *Telamoptilia prosacta*, especially in the male genitalia. However, it can be recognized by the male genitalia with the tegumen having 3−4 long setae on each lateral side, and the valva width does not change in the basal 3/4; in *Telamoptilia prosacta*, the tegumen has 9−12 long setae on each lateral side and the valva is slightly wider at middle. In the female genitalia, *Telamoptilia grewiae* sp. n. can easily be separated from all other *Telamoptilia* by the extremely short ductus bursae, not reaching the anterior margin of the seventh abdominal segment. The signum lacks a median process and is thereby more similar to signa in *Spulerina*, especially to that of *Spulerina parthenocissi* Kumata & Kuroko, 1988, than to those in *Telamoptilia*.

#### Type materials.

**Holotype**, ♂, **China:** Mt. Baxian National Nature Reserves (117°33'N, 40°11'E), 300−600 m, Ji county, Tianjin, larva coll. 6-ix-2013, *ex.* *Grewia biloba*, emerged 9-iii-2014 (indoors), leg. Tengteng Liu. **Paratypes:** 2♂, 1♀, larvae coll. 24-vi-2013, emerged 6−7-vii-2013, 1♂, 1♀, larva coll. 29-vi-2013, emerged 5-vii-2013, 4♂, 3♀, larva coll. 8-viii-2013, emerged 19−20, 22-viii-2013, 1♂, 1♀, emerged 13, 22-ix-2013, 1♂, larva coll. 12-ix-2013, emerged iv-2014 (indoors), 2♀, larva coll. 30-vi-2014, other data as holotype, genitalia slide Nos. LTT12255♀, LTT12256−7♂, LTT12261♂, LTT12555♀, LTT12556−7♂; 1♂, 300 m, 29-vi-2014, by light, leg. Kaijian Teng & Tengteng Liu, other data as holotype, genitalia slide No. LTT12561.

#### Non-type materials.

5 larvae, 25-vi-2013, 6-ix-2013, stored in ethanol, other data as holotype, BXS130628, BXS130942; 1 last instar larval skin, larva coll. 6-ix-2013, emerged 13-iii-2014, other data as holotype, mounted in Canada balsam, slide no. LTT1403L; 1 last instar larval skin, 24-vi-2013, stored in glycerine, other data as holotype, BXS130632; 2 pupal exuviae, 29-vi-2014, other data as holotype, mounted in Canada balsam, slide Nos. LTT1401−2L.

#### Host plants.

*Grewia biloba* G. Don and its variety *parviflora* Hand. –Mazz. (Malvaceae). *Telamoptilia grewiae* sp. n. is thus far the only species in Gracillariinae that is known to feed on *Grewia*. The plant family Malvaceae appears to be the main host for *Telamoptilia* with now four out of six species feeding on this family (*Telamoptilia tiliae* on *Tilia*, *Telamoptilia cathedraea* on *Urena*, *Telamoptilia geyeri* on *Pavonia*).

#### Distribution.

China (Tianjin).

#### Biology.

The larva mines on the upper surface of the host plant leaf. The mine begins as an epidermal silvery curved white line which soon enlarges to a whitish blotch. Yellowish-fuscous or fuscous lines can be found on the surface of the blotch. As the larva develops, the blotch usually incorporates the earlier linear mine. The last instar larva vacates the mine for pupation by chewing a semicircular opening near the margin of the blotch. No body colour transfer occurs in the full-grown larva of this species, compared to other *Telamoptilia* larvae which turn red when fully grown (Kumata et al. 1988). Some host plants can be seriously damaged by the mines in early September. Cocoons are usually found inlaid the leaf wrinkles, or occasionally in the corner of the rearing container. The cocoon is brown, with 2−3 brown minute bubbles on the surface. This species overwinters in pupa.

#### Etymology.

The specific name is derived from the host plant genus *Grewia*, indicating the host of the new species.

## Discussion

The forewing venation of *Telamoptilia grewiae* sp. n. is unique within the *Acrocercops* group: R_1_ and R_5_ are absent, thus R_4_ is not stalked with R_5_. The absence of R_1_ is apomorphic for *Chrysocercops* Kumata & Kuroko, 1988 (occasionally present), *Telamoptilia* and *Spulerina* (Kumata et al. 1988, Kumata 1992). Although R_1_ is also absent in *Dendrorycter* Kumata, 1978, it will not be discussed here since its status within the *Acrocercops* group is still debatable (Kumata et al. 1988). The forewing patterns and the genitalia of *Chrysocercops* are rather different from those of *Telamoptilia grewiae* sp. n. All other *Telamoptilia* and *Spulerina* have R_5_, or at least R_5_ is visible distally and stalked with R_4_ (Kumata et al. 1988). Currently there is not enough evidence for assigning the new species without R_5_ to a new genus. A comprehensive study of more species is required for further generic definition.

Kumata et al. (1988) distinguished *Telamoptilia* from *Spulerina* by the antennal scape having a minute flap, the absence of the fan-shaped comb of the valva and the signum with median process. *Telamoptilia grewiae* sp. n. has a flap as wide as the antennal scape and a signum lacking a median process, which is most similar to *Spulerina parthenocissi* as stated in the diagnosis section. Therefore the signum without median process is not an autapomorphy for *Spulerina* when defining *Telamoptilia* and *Spulerina*. The larval seta XD1 of *Telamoptilia grewiae* sp. n. is placed near the anterior margin of the prothorax shield, which resembles that of *Spulerina* Vári, but differs from that of *Telamoptilia* that has XD1 of variable placement between D1 and D2 (Kumata et al. 1988). The fully grown larva of *Telamoptilia* and *Spulerina* changes body colour into red (Kumata et al. 1988), but colour transfer does not occur in *Telamoptilia grewiae* sp. n. Consequently, the most important generic character to distinguish *Telamoptilia* from *Spulerina* is the absence of the fan-shaped comb of the valva as defined by Kumata et al. (1988), and the minute antennal scape flap and the signum with median process should be excluded for generic definition when taking *Telamoptilia grewiae* sp. n. into consideration. The position of seta XD1 and the feature of larval body colour transfer vary within *Telamoptilia*, thus should not be adopted as generic characters. Considering the unique characters of *Telamoptilia grewiae* sp. n., its phylogenetic relationship to other species of the genus *Telamoptilia* requires further study.

## Supplementary Material

XML Treatment for Telamoptilia grewiae
